# Asymptomatic Natural Human Infections With the Simian Malaria Parasites *Plasmodium cynomolgi* and *Plasmodium knowlesi*

**DOI:** 10.1093/infdis/jiy519

**Published:** 2018-10-08

**Authors:** Mallika Imwong, Wanassanan Madmanee, Kanokon Suwannasin, Chanon Kunasol, Thomas J Peto, Rupam Tripura, Lorenz von Seidlein, Chea Nguon, Chan Davoeung, Nicholas P J Day, Arjen M Dondorp, Nicholas J White

**Affiliations:** 1 Department of Molecular Tropical Medicine and Genetics, Mahidol University, Bangkok, Thailand; 2 Mahidol–Oxford Tropical Medicine Research Unit, Faculty of Tropical Medicine, Mahidol University, Bangkok, Thailand; 3 Centre for Tropical Medicine and Global Health, Nuffield Department of Medicine, University of Oxford, United Kingdom; 4 National Center for Parasitology, Entomology, and Malaria Control, Phnom Penh; 5 Provincial Health Department, Battambang, Cambodia

**Keywords:** Asymptomatic, natural, human infections, *Plasmodium cynomolgi*

## Abstract

**Background:**

In Southeast Asia, *Plasmodium knowlesi*, a parasite of long-tailed macaques (*Macaca fascicularis*), is an important cause of human malaria. *Plasmodium cynomolgi* also commonly infects these monkeys, but only one naturally acquired symptomatic human case has been reported previously.

**Methods:**

Malariometric studies involving 5422 subjects (aged 6 months to 65 years) were conducted in 23 villages in Pailin and Battambang, western Cambodia. Parasite detection and genotyping was conducted on blood samples, using high-volume quantitative PCR (uPCR).

**Results:**

Asymptomatic malaria parasite infections were detected in 1361 of 14732 samples (9.2%). Asymptomatic infections with nonhuman primate malaria parasites were found in 21 individuals living close to forested areas; *P. cynomolgi* was found in 11, *P. knowlesi* was found in 8, and *P. vivax* and *P. cynomolgi* were both found in 2. Only 2 subjects were female, and 14 were men aged 20–40 years. Geometric mean parasite densities were 3604 parasites/mL in *P. cynomolgi* infections and 52488 parasites/mL in *P. knowlesi* infections. All *P. cynomolgi* isolates had wild-type dihydrofolate reductase genes, in contrast to the very high prevalence of mutations in the human malaria parasites. Asymptomatic reappearance of *P. cynomolgi* occurred in 2 subjects 3 months after the first infection.

**Conclusions:**

Asymptomatic naturally acquired *P. cynomolgi* and *P. knowlesi* infections can both occur in humans.

**Clinical Trials Registration:**

NCT01872702.

## Teaser

Epidemiological studies in western Cambodia identified 21 subjects with asymptomatic monkey malaria parasite infections (8 with *P. knowlesi* infection and 13 with *P. cynomolgi* infection). These parasites represented 1.9% of all identified malaria parasites.

(See the Editorial Commentary by Garry on pages 511–3.)

Human infections with some monkey malaria parasites are well recognized, although the majority of reported cases have resulted from experimental infections [1]. The exception is *Plasmodium knowlesi*, a natural parasite of the long-tailed macaque (*Macaca fascicularis*) and the pig-tailed macaque (*Macaca nemestrina*), which is now the predominant cause of human malaria in Malaysia [2]. Laboratory-acquired human infections with *Plasmodium cynomolgi*, which naturally infects several monkey species, including long-tailed and pig-tailed macaques, is well described [1, 3, 4], but there is only a single case report of a natural human infection, involving a symptomatic 39-year-old female nurse in peninsular Malaysia [5].

A malaria elimination agenda has been endorsed by all countries of the Greater Mekong Subregion in Southeast Asia [6]. However, the persistence of a monkey reservoir of malaria parasites confounds malaria elimination objectives. For example, Malaysia has done very well to eliminate nearly all human malaria but is left now with residual *P. knowlesi* in the monkey population as a source of human infections [6]. Submicroscopic malaria parasite infections [7] are important contributors to malaria endemicity in areas of generally low seasonal transmission [8, 9]. In the course of conducting malariometric surveys in western Cambodia, we discovered asymptomatic human infections with both *P. knowlesi* and *P. cynomolgi* and present here a molecular evaluation of these infections.

## MATERIALS AND METHODS

### Sample Sites and Locations

**Figure 1.**
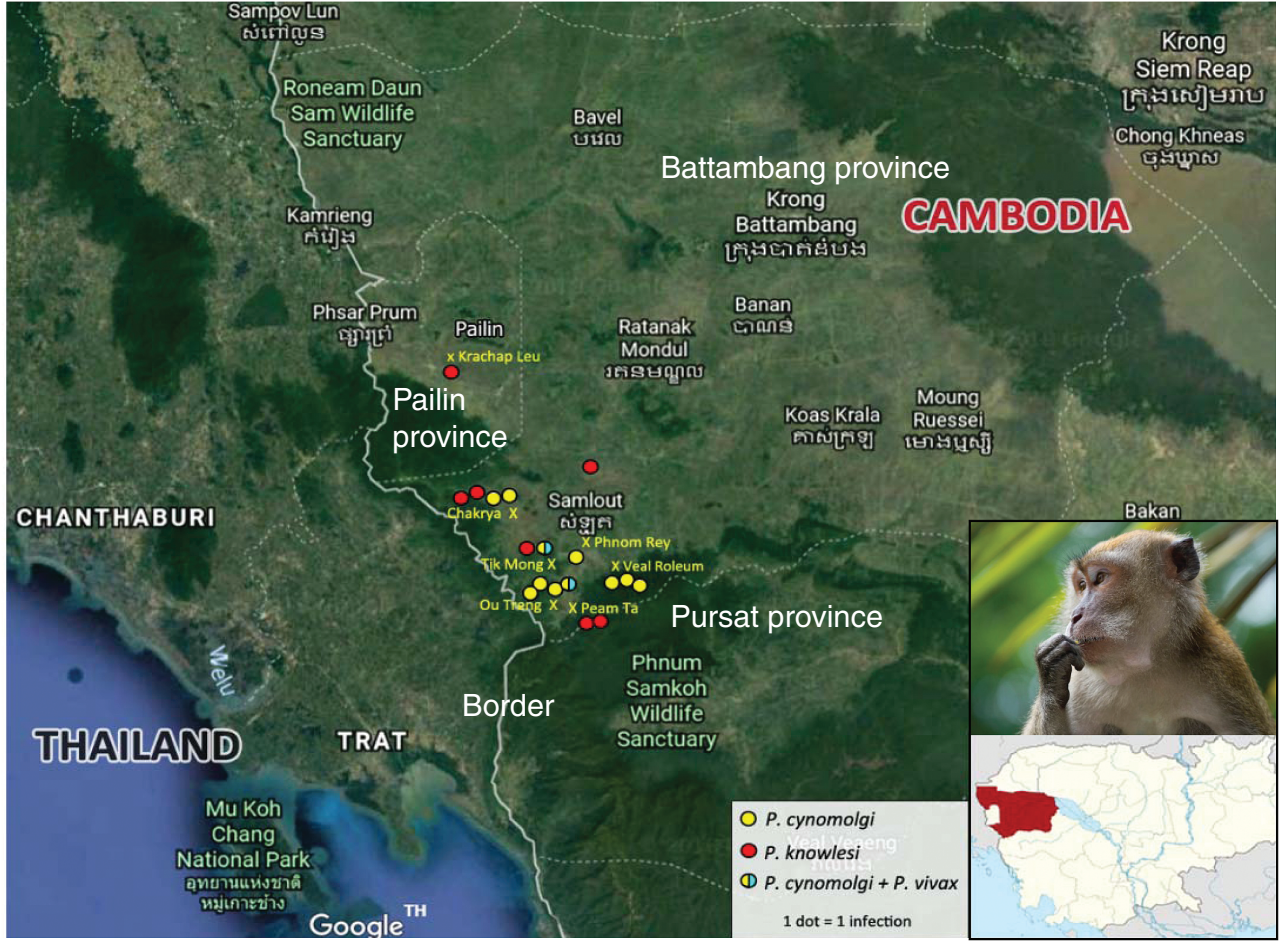
Locations of subjects asymptomatically infected with *Plasmodium cynomolgi* and *Plasmodium knowlesi*. The inset (bottom right) shows the natural host, *Macaca fascicularis* (above), and the area in Cambodia under study (below).

Malariometric surveys were conducted in 23 villages in the Pailin and Battambang provinces of western Cambodian as described previously [8, 10, 11] (Figure 1). In brief, after extensive community engagement and obtaining individual informed consent, anthropometric and demographic data were collected, including tympanic temperature, height, weight, health status, malaria history, bed net use, travel history, and occupation. In addition, a 1.5-mL venous blood specimen was collected in tubes containing ethylenediaminetetraacetic acid and kept cool until storage at −70°C. If a participant was febrile (tympanic temperature, ≥37.5°C), a malaria rapid diagnostic test was performed (SD Bioline Malaria Ag P.f/P.v, Standard Diagnostics, Gyeonggi-do, Republic of Korea), and if the result was positive, treatment was provided by the village malaria worker according to national guidelines. In brief, during 2013–2014 in 3 villages in Pailin Province and during 2015–2016 in 5 villages in Battambang Province, all residents aged 6 months to 65 years were invited to participate in cross-sectional surveys conducted over a 12-month period. From March to May 2015, 1000 residents of 20 villages in Battambang were surveyed using a stratified sampling method to obtain approximately equal numbers of adults aged ≥18 years from 4 groups (men aged <30 years, men aged >30 years, women aged <30 years, and women aged >30 years).

Of 6100 invited participants, 5422 provided a blood sample at least once. A total of 14732 samples were sent to the reference laboratory in Bangkok for testing by high-volume quantitative PCR (uPCR) targeting the gene encoding *Plasmodium* genus–specific 18S ribosomal RNA (rRNA) as described previously. This has a lower limit of accurate quantification of 22 genomes/mL [12]. Ethics approval was obtained from the Cambodian National Ethics Committee for Health Research (reference numbers 012, 29.01.2014; 042, 20.02.2015; and 051, 18.02.2016) and the Oxford Tropical Research Ethics Committee (reference number 1017-13). The collecting, processing, and handling of venous blood samples were performed in accordance with the Appropriate Technology in Health (PATH) guidelines.

### *P. cynomolgi* and *P. knowlesi* Molecular Identification

Samples testing positive by uPCR were then assessed by real-time PCR, using species-specific probes for detection of the 5 human malaria parasites: *Plasmodium falciparum, Plasmodium vivax, Plasmodium malariae*, and the 2 *Plasmodium ovale* species. A subset of samples containing *Plasmodium* DNA did not amplify with these species-specific probes and were investigated further.

We developed and validated a nested PCR targeting the gene encoding the 18S rRNA of the following malaria parasites: *P. cynomolgi*, *P. knowlesi, Plasmodium coatneyi, Plasmodium brasilianum*, *Plasmodium inui*, *Plasmodium simium, Plasmodium semiovale, Plasmodium fieldi, Plasmodium fragile, Plasmodium vinckei, Plasmodium yoeli, Plasmodium chabaudi, Plasmodium berghei,* and *Plasmodium adleria* (Supplementary Materials). Two pairs of oligonucleotide primers were designed to amplify both the A and S types of the genes encoding 18S rRNA (Supplementary Table 1). Nested PCR was performed for 25 cycles during the first round and for 30 cycles during the second round, using the following conditions: 1 minute at 94°C, 1 minute (for annealing) at 53°C, and 1 minute (for extension) at 72°C. The PCR products (233–298 bp) were then sent to a commercial laboratory for DNA sequencing (Macrogen, South Korea). The sequencing results were aligned against the genes encoding the 18S rRNA of primate malaria parasite reference strains (accession numbers are in the Supplementary Table 1). The analysis was performed with Bioedit software (Abbott, CA).

### Whole-Genome Amplification

For further analysis of very-low-density infections with *P. cynomolgi* (n = 4) and *P. knowlesi* (n = 3), we performed whole-genome amplification, using multiple displacement amplification by Phi29 (Repli-g Minikit; Qiagen, Germany) according to the manufacturer’s instructions. Following collection, white blood cells were depleted from blood samples so that contaminating human DNA was less likely to limit the amplification efficiency in this system. To rule out cross-contamination during processing, 1 negative control consisting of only water was added for every 3 samples. We evaluated and compared DNA sequences and microsatellites before and after amplification in 14 samples, of which 9 were positive for *P. cynomolgi*, and 5 were positive for *P. knowlesi*. Findings were 100% concordant.

### Assessment of the Gene Encoding Dihydrofolate Reductase–Thymidylate Synthase (*dhfr-ts*)

Point mutations in *P. cynomolgi* and *P. knowlesi dhfr-ts* were assessed by nested PCR amplification covering the full length of *dhfr-ts* (approximately 1800 bp, including 1 exon), followed by sequencing of the gene in an ABI Sequencer (Macrogen, Korea). The sequences were then aligned against the *dhfr-ts* of reference Cambodian strain (PCYB_053150) of *P. cynomolgi* and strain H (PKNH_0509600) of *P. knowlesi* and were analyzed using Bioedit software (Abbott, CA).

### *P. cynomolgi* and *P. knowlesi* Microsatellite Genotyping and Cluster Analysis

To assess genetic relatedness between infecting nonhuman primate malaria parasites and between initial and recurrent infections, microsatellite genotyping was performed. For *P. cynomolgi*, 8 microsatellite markers of di/tri/tetra-nucleotide repeats distributed across chromosomes 1–12, named 1.307 (TCT), 2.36 (AT), 3.574(GTGA), 4.41(TCT), 4.462(TCC), 5.956(AAG), 6.455(AGG), 7.1006(ATAC), 10.179(TA), 10.621(TGTA), 11.1096(TGTA), and 12.286(AAT), were assessed using seminested PCR as described previously [13]. For *P. knowlesi,* 10 microsatellite markers of trinucleotide repeats spanning chromosomes 3–13, named NC03_2(AAG), CD05_06(TAA), CD08_61(TAC), NC09_1(GAA), NC10_1(TTA), CD11_157(GAG), NC12_2(AAT), NC12_4(GAA), CD13_61(AAC), and CD13_107(AGG), were assessed using seminested PCR as described previously [14]. The lengths of the PCR-generated products were measured in comparison to internal size standards (Genescan 500 LIZ) on an ABI 3100 Genetic analyzer (PE Applied Biosystems), using Genescan and Genotyper software (Applied Biosystems) to measure allele length and quantify peak heights. Samples that amplified poorly for particular loci (maximum peak height, <300 fluorescent units) were reamplified. Negative control samples containing no template were included in each amplification run, to rule out cross-contamination.

Genetic similarity between isolates was analyzed and a dendrogram constructed on the basis of the microsatellite markers, using an unweighted pair group method with arithmetic mean (UPGMA), performed with BioNumerics software, version 7.5 (Applied Maths, Belgium).

This trial was registered on 4 June 2013 at Clinicaltrials.gov (NCT01872702).

## RESULTS

Of the 14732 samples collected (7320 from Battambang and 7412 from Pailin), uPCR indicated that 1361 (9.2%; 663 from Battambang and 698 from Pailin) contained malaria parasite DNA. Of these, DNA in 1125 could be identified to the species level: *P. falciparum* was detected in 114 (10.1%), *P. vivax* was detected in 976 (86.8%), and both species were detected in 14 (1.2%) [8, 10, 11] (Table 1). In the initial batch of testing for *P. vivax* species, using a less sensitive and specific real-time PCR protocol [15], 853 samples were found to contain *P. vivax* DNA. However, because of the 98% similarity between the genes encoding the 18S RNA of *P. vivax* and *P. cynomolgi*, which raised the possibility of misidentification, we subsequently retested the same set of samples, using the specific primate nested PCR and sequencing protocol. Three of 853 specimens (0.3%) originally identified as containing *P. vivax* actually contained *P. cynomolgi* only.

**Table 1.** Asymptomatic Malaria Parasite Infections in Western Cambodia

Study Location	Study Interval	Survey Samples, No.	Specimens With Positive uPCR Results, No.	Specimen Positivity for Parasites, No.			Specimens With Positive Real-time PCR Results, No.			
				*P. cynomolgi*	*P. cynomolgi + P. vivax*	*P. knowlesi*	*P. vivax*	*P. falciparum*	Mixed^a^	Not Identified
Pailin	Jun 2013–Jun 2014	7412	698	0	0	1	445	58	9	185
Battambang	Mar 2015–May 2015	1000	91	0	0	1	78	7	0	5
Battambang	July 2015–Nov 2016	6320	572	11	2	6	453	49	5	46
Overall	…	14732	1361	11	2	8	976	114	14	236

Abbreviations: PCR, polymerase chain reaction; *P. cynomolgi*, *Plasmodium cynomolgi*; *P. falciparum*, *Plasmodium falciparum; P. knowlesi*, *Plasmodium knowlesi*; *P. vivax, Plasmodium vivax*; uPCR, high-volume quantitative PCR.

^a^Positive for both *P. vivax* and *P. falciparum*.

### Asymptomatic *P. cynomolgi* Human Infections

In total, 11 *P. cynomolgi* monoinfections were found in asymptomatic afebrile residents from 5 villages located close to the forest. All individuals were male; the median age was 28 years (range, 7–64 years). The 5 villages comprised Chakrya, Ou Treng, Tik Mong, Veal Roleum, Phnom Rey, in Battambang, and Krong Pailin, in Pailin, Western Cambodia (Figure 1). The geometric mean parasite genome density was 3604 parasites/mL (range, 578–26923 parasites/mL; Figure 3). Mixed infections with *P. cynomolgi* and *P. vivax* were found in 2 additional subjects. Thus, the overall prevalence of asymptomatic human *P. cynomolgi* infection was approximately 2 cases per 1000 persons. *P. cynomolgi* comprised approximately 1% of the malaria parasites isolated. Recurrences of *P. cynomolgi* infection were identified approximately 3 months after initial isolation in 2 individuals, from Charkrya and Ou Treng.

### Asymptomatic *P. knowlesi* Human Infections

Eight *P. knowlesi* monoinfections were found in 4 villages (Chakrya, Peam Ta, Tik Mong, and Samlout, in Batambang, and Krachap Lue, in Pailin; Figure 1), using nested PCR, and were confirmed by DNA sequencing results. Two subjects were females, and 6 were males; the median age was 35.5 years (range, 23–58 years). All individuals were asymptomatic, but 2 had a tympanic temperatures of 37.4°C. The geometric mean parasite genome density was 52488 parasites/mL (range, 336–1306852 parasites/mL; Figure 3). Persistent or recurrent *P. knowlesi* infection was not found in this study, and, apart from *P. cynomolgi,* no other nonhuman-primate malaria parasites were identified. Of the 21 subjects with simian malaria parasite infections, only 2 were females (whereas females comprised 29% of subjects with asymptomatic human malaria parasite infections; *P* = .08), and 14 were men aged 20–40 years, reflecting the demographic group most likely to enter and spend time in or near the forest.

### Characterization of *dhfr-ts* Genes

The full length *dhfr-ts* gene (approximately 1884 bp) was sequenced in all *P. cynomolgi* infections. No nonsynonymous mutations were found. Thus, 0 of the 7 drug resistance point mutations in the *dhfr* drug binding pocket, which are highly prevalent in *P. vivax* isolates in this area [16–18], were found, providing further evidence that these were indeed primate parasites. Only a single nonsynonymous mutation (S231N) in the TS gene was identified. Interestingly, all 13 isolates of *P. cynomolgi* contained identical repeats within the *Pcdhfr* of (SGDTH)(SGDNTNG)(SGDAVGT). This contrasts with *P. vivax*, in which the number of intragenic repeats is highly variable. In the 2 *P. cynomolgi* and *P. vivax* mixed infections, only the *P. vivax dhfr-ts* was amplified by PCR, presumably because *P. cynomolgi* was the minority population. The *P. vivax dhfr-ts* had the common double resistance mutations at S58R and S117N with the (GGDNTS)(GGDNADK) repeat.

The full length of *dhfr-ts* gene (approximately 1881-bp) sequences of the 8 *P. knowlesi* isolates had no nonsynonymous mutations, although there was 1 deletion at codon 105 without any interruption of protein translation. There was also 1 nonsynonymous mutation (G257S) in the TS gene.

### Microsatellite Markers, Genetic Variation of *P. cynomolgi*, and Genotyping of Pathogens Involved in Recurrent Infections

**Figures 2.**
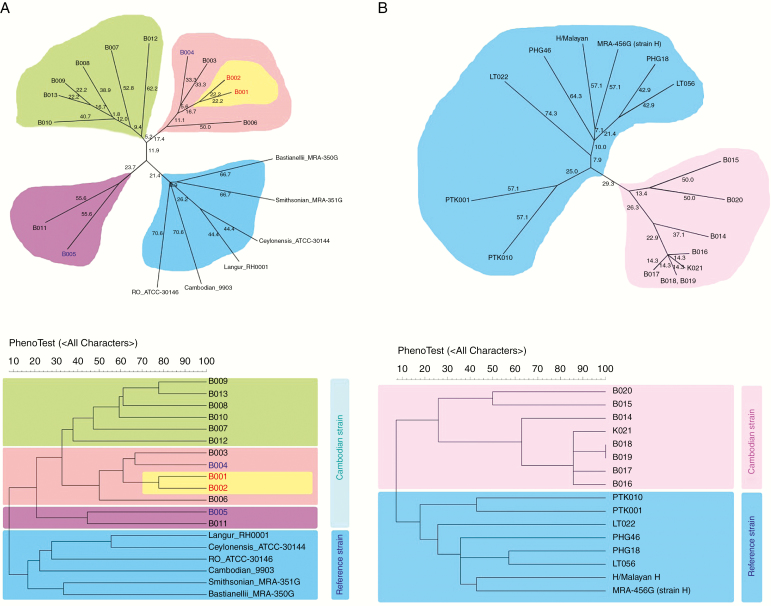
Cluster analysis *of Plasmodium cynomolgi* isolates (*A*) and *Plasmodium knowlesi* isolates (*B*). *A*, Dendrogram of the interstrain relatedness of *P. cynomolgi* obtained from 13 asymptomatic subjects in Cambodia, including 2 pairs of primary and recurrent infections. Microsatellite types were compared to reference strains [13]. Cluster analysis was based on typing of 8 microsatellites, using genetic similarity indexes obtained by the unweighted pair group method arithmetic averages (UPGMA). The analysis revealed a cluster within the Cambodian infections and 1 very related parasite pair (initial and recurrent infection). Another pair clearly diverged between the primary and recurrent infection. *B*, Dendrogram based on microsatellite typing of *P. knowlesi* obtained from 8 asymptomatic subjects in Cambodia, compared with reference strains [14]. Cluster analysis used the same UPGMA method. The analysis revealed a cluster within the Cambodian infections that clearly diverged from reference strains. The upper panels show an unrooted tree, and the lower panels show a rooted tree.

To characterize the diversity of the *P. cynomolgi* isolates, 8 tandem repeat loci bearing 2–4 bp specific for *P. cynomolgi* were tested (n = 13). The mean number (±SE) of alleles per locus was 6.37 ± 0.29 (Supplementary Table 4). Diversity, or mean expected heterozygosity, was quite high (range, 0.495–0.868; mean [±SE], 0.68 ± 0.017). The mean multiplicity of infection (±SE) was 1.28 ± 0.036. Microsatellite typing and subsequent cluster analysis by UPGMA included the previously published microsatellite genetic patterns of 7 reference *P. cynomolgi* strains for comparison (Figure 2A). The dendrogram indicated that the *P. cynomolgi* strains infecting humans in Cambodia were clustered and that the 7 previously isolated strains clearly diverged from these Cambodian isolates.

**Figure 3.**
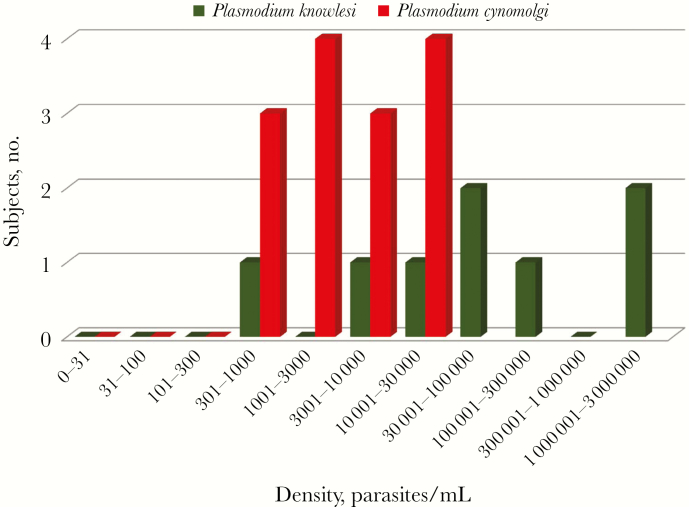
Parasite genome densities of *Plasmodium cynomolgi* and *Plasmodium knowlesi* in asymptomatic human infections observed in Cambodia between 2013–2016.

Two of the 14 subjects (14.2%) presented with recurrent *P. cynomolgi* infections 3 months after the initial infection. One of the 2 isolate pairs was closely related genetically, suggesting that they were sibling parasites derived from the same inoculation. This could result either from continued blood-stage parasite infection with fluctuating densities of the sibling parasites or from relapse. Isolates from the other pair were clearly genetically heterologous, suggesting a heterologous relapse or possibly reinfection.

### Microsatellite Markers and Genetic Variation of *P. knowlesi*

The diversity of the *P. knowlesi* parasite isolates was assessed using 7 microsatellite markers. The mean number (±SE) of alleles per locus was 2.85 ± 0.17 (Supplementary Table 5). Diversity, or mean expected heterozygosity, was low (mean [±SE], 0.44 ± 0.02). The mean multiplicity of infection (±SE) was 1.05 ± 0.014. Cluster analysis by UPGMA incorporated previously published microsatellite genetic patterns of 7 *P. knowlesi* strains. The dendrogram indicated that the *P. knowlesi* in Cambodia were clustered and clearly diverged from the reference laboratory strains recovered many years ago (Figure 2B).

## DISCUSSION

This is the first report from a malaria-endemic area of asymptomatic infection with the nonhuman-primate malaria parasite *P. cynomolgi.* This parasite was first discovered by Halberstadter and von Prowazek, in Java in 1907, in blood specimens collected from a cynomolgus monkey [19], also known as the crab-eating or long-tailed macaque (*M. fascicularis*). *P. cynomolgi* in rhesus monkeys was recognized as behaving similarly to *P. vivax*, with frequent relapses from an exoerythrocytic source (later shown to be the dormant “hypnozoites” in the liver), and so this infection became the animal model for relapsing malaria. The potential for mosquito-to-human transmission of *P. cynomolgi* was illustrated first by the renowned entomologist Don Eyles when he and then his assistant became ill with malaria while studying mosquito transmission in laboratory primates [1, 20]. Before then, it was thought that humans could not be infected naturally with primate malaria parasites. Many artificial human infections have since been studied [1, 3, 4]. Morphologically, genetically, and biologically *P. cynomolgi* is very similar to *P. vivax,* although the incubation period of sporozoite-induced infections in humans is significantly longer [1]. *P. cynomolgi* has not caused severe malaria in experimentally infected humans. Studies of untreated infections with both the M and B strains by Coatney et al documented persistence of the initially symptomatic *P. cynomolgi* human infections for as long as 58 days [1, 3, 4]. Thus, as with the other malaria parasites, persistence in blood can follow symptomatic infection, or in all probability newly acquired infections may be asymptomatic, particularly if there has been previous exposure. There is serological cross-reactivity, but whether previous exposure to *P. vivax* results in *P. cynomolgi* premunition, or vice versa, is not known. Important anopheline vectors of human malaria in Cambodia and Vietnam (*Anopheles dirus* and *Anopheles maculatus*) have been shown to carry *P. cynomolgi* and *P. knowlesi* sporozoites [21, 22]. The main vector in these asymptomatic Cambodian human *P. cynomolgi* infections is likely to be the highly effective forest vector *A. dirus*.

The morphological and genetic similarities of *P. cynomolgi* and *P. vivax* probably contribute to underrecognition of the monkey parasite in human infections in the Greater Mekong Subregion. Symptomatic patients in whom malaria is diagnosed either by microscopy or a genus-specific rapid diagnostic test will probably be labeled as having vivax malaria. In malariometric surveys, cross-reactivity in some of the PCR methods used may also lead to misdiagnosis, as happened early in this study. The specificity of the PCR method finally used in this study is supported by the analysis of the *dhfr-ts* genes. Their protein product is the target of antifolate drugs. Nonsynonymous drug resistance mutations are found in nearly all *P. vivax* (and *P. falciparum*) isolates in Cambodia and adjoining Thailand [16–18], yet none of the *P. cynomolgi* isolates contained the corresponding *Pcdhfr* mutations. This is explained by the absence of drug pressure in the primate population and the likely very small contribution of human infection to *P. cynomolgi* transmission and epidemiology.

In 2 subjects, *P. cynomolgi* was recovered twice, with an intervening interval of approximately 3 months. In one case genetically distinct parasites were identified, and in the other the paired parasites were related, indicating that they were siblings derived from the same mosquito inoculum. Whether these were relapses, reinfections, or persistent infections with blood-stage parasites, with a mixed infection, cannot be determined with certainty, although reinfection is highly unlikely in the latter case. The genetic diversity of the *P. cynomolgi* and *P. knowlesi* isolates was low. The multiplicity of *P. cynomolgi* infections (mean [±SE], 1.28 ± 0.036) was lower than observed in *P. vivax* infections from Thailand (mean, 2.67), Myanmar (mean, 2.68), and India (mean, 1.84), which may reflect the relative rarity of successful transmission to humans [23]. These simian parasites recovered from humans may be generally representative of those circulating in the monkey population, or they could represent a subset that more readily infect humans. *P. cynomolgi* human red blood cell invasion is restricted to reticulocytes expressing both transferrin receptor 1 (Trf1 or CD71) and the Duffy antigen/chemokine receptor (DARC) or CD234 [24], so the possibility that the *P. cynomolgi*–infected individuals possess a facilitatory genetic polymorphism affecting these receptors also cannot be excluded.

Naturally acquired *P. knowlesi* infections are increasingly recognized in Southeast Asia—particularly in Malaysia [2, 25–30]. Close proximity to the natural hosts, long-tailed and pig-tailed macaques, is the main risk factor. Humans and monkeys are often bitten by the same anopheline mosquito vectors [21, 22]. Interestingly, in Sabah and Sarawak, *P. knowlesi* is the main cause of human malaria, yet *P. inui* and *P. cynomolgi* are the more prevalent malaria parasites in long-tailed macaques, which are the main local *P. knowlesi* reservoir [31, 32]. Asymptomatic human *P. cynomolgi* infections have not been found there to date [28]. This may be related to intraspecies parasite differences in their infectivity to human. Vector differences are an unlikely explanation, given the relatively common transmission of *P. knowlesi* by the same anophelines. *P. knowlesi* is a quotidian parasite that may reach high densities in humans and cause potentially lethal infections, although the majority of presentations are associated with uncomplicated malaria and low-density infections [2]. With the advent of sensitive PCR methods of detection, asymptomatic infections have been documented across the region [3, 25–30]. *P. knowlesi* may be mistaken for *P. falciparum* at the young ring stage and with *P. malariae* at the trophozoite stage. *P. knowlesi* is detected by pan-species rapid tests, although the limit of detection is higher than for the human malaria parasites. In early PCR protocols, there was cross-reactivity with *P. vivax*, its closest phylogenetic relative among the human malaria parasites, but more-recent methods such as those used here are highly specific. As with *P. cynomolgi, P. knowlesi* is not under antimalarial drug selection pressure and so remains fully susceptible to all of the antimalarial drugs [33]. Although the number of subjects with asymptomatic nonhuman-primate malaria parasite infections recorded in this large survey was small, there was still a significant difference in the calculated parasite densities, with *P. cynomolgi* carried at lower parasitemia levels. Such densities, if maintained, might be too low to infect anopheline mosquitoes.

In malariometric surveys that employ microscopy, rapid diagnostic tests or non–species-specific PCR methods of detection, the nonhuman primate malaria parasites will not be recognized. With species-specific PCR protocols, positive samples that are not recognized by the human malaria parasite–specific probes are likely to carry nonhuman-primate malaria parasites. If there is insufficient DNA for accurate characterization, whole-genome amplification protocols may be used successfully, as in this study. If there is any uncertainty, then genes other than that encoding 18S RNA can be assessed. In this study, the finding of a “wild-type” *dhfr* in an area where nearly all human malaria parasites are mutated at this locus provides strong supportive evidence.

As human malaria is controlled and the prevalence falls, primate malaria parasites will comprise an increasing proportion of cases in areas where monkeys live in close proximity to people. In this study, conducted in western Cambodia, nonhuman-primate malaria parasites comprised 1.9% of identified parasites. Regional malaria elimination assessments must recognize the propensity of these primate malaria parasites to cause asymptomatic infection, as well as symptomatic infection.

## Supplementary Data

Supplementary materials are available at *The Journal of Infectious Diseases* online. Consisting of data provided by the authors to benefit the reader, the posted materials are not copyedited and are the sole responsibility of the authors, so questions or comments should be addressed to the corresponding author.

Supplementary Table1-5

## Notes

***Author contributions.*** M. I., N. P. J. D., A. M. D., and N. J. W. contributed to study design. T. J. P., R. T., L. V. S., C. N., C. D., and A. M. D. collected clinical samples and data. W. M., K. S., C. K., and M. I. did the molecular laboratory work and analysis. M. I., T. J. P., R. T., L. V. S., N. P. J. D., A. M. D., and N. J. W. analyzed the data. M. I. and N. J. W. wrote the report. All authors read and approved the final manuscript.

***Acknowledgments.*** We thank Mehul Dhorda, Cholrawee Promnarate, Im Sambo, and Pasathorn Sirithiranont, for sample and data management.

***Financial support.*** This work was supported by the Bill & Melinda Gates Foundation (award number OPP1081420), and was conducted as part of the Wellcome Trust–Mahidol University–Oxford Tropical Medicine Research Programme, which is funded by the Wellcome Trust of Great Britain (award number 101148/Z/13/Z). The study sponsor is University of Oxford, United Kingdom.

***Potential conflicts of interest.*** All authors: No reported conflicts of interest. All authors have submitted the ICMJE Form for Disclosure of Potential Conflicts of Interest. Conflicts that the editors consider relevant to the content of the manuscript have been disclosed.
