# Supplementary material for: Asymptomatic Natural Human Infections With the Simian Malaria Parasites Plasmodium cynomolgi and Plasmodium knowlesi
Source: J Infect Dis. 2018 Oct 8;219(5):695–702. doi: 10.1093/infdis/jiy519 (PMC6376906; doi:10.1093/infdis/jiy519)
Supplement: Supplementary Table1-5 [file jiy519_suppl_supplementary-table1-5.docx]

**Supplementary Table 1:** Identity (%) of full length 18srRNA gene between A type and S types of the primate malarias

**Supplementary Table 2:** Oligonucleotide primers, and PCR protocol targeted of both A and S types 18srRNA of malaria parasites*,* which covered *Plasmodium cynomolgi*, *Plasmodium knowlesi, Plasmodium coatney, Plasmodium brasilianum*, *Plasmodium inui*, *Plasmodium simium, Plasmodium semiovale, Plasmodium fieldi, Plasmodium fragile, Plasmodium vinckei, Plasmodium yoeli, Plasmodium chabaudi, Plasmodium berghi,* and *Plasmodium adleria.*

| **Primate Malaria** | **Primers** | **Sequences (5’>3’)** | **Final concentration** | **Temperature and cycling profiles** | **Product**  **Size (bp)** |
| --- | --- | --- | --- | --- | --- |
| Nest1 | PlasmoM_N1F  PlasmoM_N1R | ATGGCCGTTTTTAGTTCGTG  TTGTGTTAGACACACATCGTTCC | 1X Buffer, 3 mM MgCl2, 250 uM dNTPs, 250 nM Primers, 0.4 U Taq DNA polymerase (5 U/ul) | 1 minute at 94°C, 1 minute annealing at 53°C, and 1 minute extension at 72°C, N1: 25 cycles, N2: 30 cycles |  |
| Nest2 | PlasmoM_N2F  PlasmoM_N1R | GTTAATTCCGATAACGAACGAGA  TTGTGTTAGACACACATCGTTCC |  |  | 233-298 bp |
| Sequencing primer | PlasmoM_N2F  PlasmoM_N1R | GTTAATTCCGATAACGAACGAGA  TTGTGTTAGACACACATCGTTCC |  |  |  |

**Supplementary Table 3:** Number of samples was collected per individual.

| Number of times individuals were sampled | Number of subjects | |
| --- | --- | --- |
|  | Battambang | Pailin |
| 1 | 908 | 553 |
| 2 | 232 | 314 |
| 3 | 255 | 323 |
| 4 | 333 | 378 |
| 5 | 562 | 508 |
| 6 | 8 | 71 |
| 7 | 4 | 13 |
| 8 | 9 | 12 |
| 9 |  | 11 |
| 10 |  | 10 |
| 11 |  | 18 |
| 12 |  | 51 |
| Median (range) | 3 (1-9) | 3 (1-12) |

Supplementary Table 4. *Microsatellite genotyping of P. cynomolgi*

|  |  |  |  | Eight microsatellite Markers | | | | | | | |
| --- | --- | --- | --- | --- | --- | --- | --- | --- | --- | --- | --- |
| No. | Sample ID | Host | Village | 1.307 | 2.36 | 4.41 | 4.462 | 5.956 | 6.455 | 7.1006 | 10.621 |
|  |  |  |  | (TCT) | (AT) | (TCT) | (TCT) | (AAG) | (GAA) | (ATCT) | (TGTA) |
| Reference stain | Bastianellii_MRA-350G | Macaca fascicularis |  | 295 | 195 | 267 | 244 | 192 | 200 | 272 | 269 |
|  | Berok_446 | Macaca nemestrina |  | 280 | 172 | 270 | 232 | 144 | 194 | 288 | 221 |
|  | Cambodian_9903 | Macaca fascicularis |  | 307 | 170 | 273 | 235 | 198 | 203 | 224 | 261 |
|  | Ceylonensis_ATCC-30144 | Macaca sinica |  | 307 | 205 | 267 | 238 | 213 | 203 | 272 | 230 |
|  | Langur_RH0001 | Presbytis entellus thersites |  | 292 | 205 | 267 | 229 | 198 | 203 | 324 | 230 |
|  | Smithsonian_MRA-351G | Macaca speciosa |  | 274 | 170 | 270 | 244 | 192 | 191 | 264 | 265 |
|  | RO_ATCC-30146 | Macaca mulatta |  | 292 | 212 | 273 | 247 | 198 | 197 | 272 | 265 |
| 1 | B001 | Homo sapiens | Chakrya | 335/443 | 207 | 276 | 394 | 213 | 195 | 228 | 270 |
| 2 | B002 | Homo sapiens | Chakrya | 335 | 229 | 276 | 235 | 213 | 195 | 228 | 270 |
| 3 | B003 | Homo sapiens | Ou Treng | 275/335 | 147/207 | 276 | 235 | 159/ 192/ 249 | 207/ 294 | 228 | 270 |
| 4 | B004 | Homo sapiens | Ou Treng | 335 | 131 | 276 | 235 | 183 | 204 | 228 | 270 |
| 5 | B005 | Homo sapiens | Ou Treng | 335 | 147/191 | 192/240 | 232 / 292/ 394 | 165 /192 | 327 | 192/256/264 | 262 |
| 6 | B006 | Homo sapiens | Ou Treng | 239/374/443 | 207 | 276 | 184/ 223/ 235 / 292/ 394 | 192 | 327 | 228 | 270 |
| 7 | B007 | Homo sapiens | Tik Mong | 266/443 | 201 | 285 | 235 | 159/ 192/ 249 | 201 | 228 | 262 |
| 8 | B008 | Homo sapiens | Tik Mong | 338 | 229 | 261 | 235 | 213 | 204 | 228 | 262 |
| 9 | B010 | Homo sapiens | Veal Roleum | 275/308 | 201 | 261 | 235 | 213 | 207 | 232 | 262 |
| 10 | B009 | Homo sapiens | Veal Roleum | 275/305/335 | 201 | 261 | 235 | 213 | 204 | 228 | 266 |
| 11 | B011 | Homo sapiens | Phnom Rey | 350 | 201 | 279 | 184 /292 /310/ 394 | 192 | 327 | 192 | 262 |
| 12 | B012 | Homo sapiens | Ou Treng | 266/287 | 201 | 261 | 226/394 | 192 | 204 | 228 | 266 |
| 13 | B013 | Homo sapiens | Veal Roleum | 308 | 201 | 261 | 235 | 213 | 201 | 228 | 266 |
| No. of allele | | | | 11 | 6 | 6 | 8 | 6 | 6 | 5 | 3 |
| Heterozygosity (He) | | | | 0.782 | 0.769 | 0.744 | 0.59 | 0.615 | 0.846 | 0.41 | 0.705 |
| Multiplicity of Infection (MOI) | | | | 1.692 | 1.154 | 1.077 | 1.769 | 1.385 | 1.077 | 1.154 | 1 |

Supplementary Table 5. *Microsatellite genotyping of P. knowlesi*

| No. | Sample ID | Host | Village | Microsatellite Markers | | | | | | |
| --- | --- | --- | --- | --- | --- | --- | --- | --- | --- | --- |
|  |  |  |  | NC03_2 | CD05_06 | NC09_1 | NC10_1 | CD11_157 | NC12_2 | CD13_107 |
|  |  |  |  | AGG | TAA | GAA | TTA | GAG | AAT | AGG |
| Reference strain | H/Malayan H |  |  | 153 | 249 | 288 | 274 | 247 | 346 | 185 |
|  | MRA-456G (stain H) |  |  | 129 | 248 | 276 | 265 | 248 | 337 | 186 |
| Divis PCS et al., 2015 | LT022 | Macaca fascicularis |  | 129 | 257 | 284 | 258 | 248 | 319 | 189 |
|  | LT056 | Macaca fascicularis |  | 132 | 248 | 284 | 267 | 248 | 319 | 189 |
|  | PTK001 | Macaca nemestrina |  | 132 | 248 | 287 | 264 | 257 | 322 | 183 |
|  | PTK010 | Macaca nemestrina |  | 132 | 245 | 287 | 258 | 260 | 316 | 183 |
|  | PHG46 | Homo sapiens |  | 135 | 245 | 287 | 267 | 248 | 358 | 186 |
|  | PHG18 | Homo sapiens |  | 132 | 248 | 278 | 267 | 248 | 355 | 186 |
| 1 | B016 | Homo sapiens | Peam Ta | 129 | 245 | 276 | 262 | 238 | 337 | 198 |
| 2 | B018 | Homo sapiens | Tik Mong | 129 | 245 | 276 | 262 | 238 | 343 | 198 |
| 3 | B014 | Homo sapiens | Chakrya | 126 | 245 | 276 | 262 | 238 | 343 | 213 |
| 4 | B019 | Homo sapiens | Tik Mong | 129 | 245 | 276 | 262 | 238 | 343 | 198 |
| 5 | B017 | Homo sapiens | Peam Ta | 129 | 245 | 276 | 262 | 238 | 325 | 198 |
| 6 | B015 | Homo sapiens | Chakrya | 129 | 212 | 276 | 262 | 142 | 106 | ND |
| 7 | K021 | Homo sapiens | Krachap Lue | 129 | 245 | 276 | 262 | 238 | 331 | 198 |
| 8 | B020 | Homo sapiens | Samlout | 126 | 212 | 108/123 | 208/184/154 | 142 | 106 | 213 |
| No. of alleles | | | | 2 | 2 | 3 | 4 | 2 | 5 | 2 |
| Heterozygosity (He) | | | | 0.429 | 0.429 | 0.25 | 0.25 | 0.429 | 0.857 | 0.476 |
| Multiplicity of Infection (MOI) | | | | 1 | 1 | 1.125 | 1.25 | 1 | 1 | 1 |
